# Exploring nutritional risks of the specific carbohydrate diet: food and nutrient intake in children with juvenile idiopathic arthritis

**DOI:** 10.1017/jns.2024.92

**Published:** 2025-01-23

**Authors:** Naima Hagström, Afsaneh Koochek, Eva Warensjö Lemming, Anders Öman, Henrik Arnell, Lillemor Berntson

**Affiliations:** 1 Department of Women’s and Children’s Health, Uppsala University, Uppsala, Sweden; 2 Department of Food Studies, Nutrition and Dietetics, Uppsala University, Uppsala, Sweden; 3 Department of Surgical Sciences, Medical Epidemiology, Uppsala University, Uppsala, Sweden; 4 Department of Pediatric Gastroenterology, Hepatology and Nutrition, Astrid Lindgren Children’s Karolinska University Hospital, Stockholm, Sweden; 5 Department of Women’s and Children’s Health, Karolinska Institutet, Stockholm, Sweden

**Keywords:** Dietary assessment, Diet therapy, Food intake, Juvenile idiopathic arthritis, Specific carbohydrate diet

## Abstract

Diet is considered a key research priority for juvenile idiopathic arthritis (JIA), garnering considerable interest from affected families. Despite this, research studies focusing on dietary interventions remain scarce. The specific carbohydrate diet (SCD) has shown potential, however, its nutritional consequences and risks are not well understood. This study aims to describe and evaluate food and nutrient intakes in children with JIA adhering to the SCD and contextualize the results relative to recommendations and intakes in the general population. In a secondary analysis, food and nutrient intakes from three-day dietary records of ten children, following a four-week SCD intervention, were evaluated against the Nordic Nutrition Recommendations 2023 and Riksmaten Adolescents data (RMA) (n = 1282). All children following the SCD met the recommended minimum intake of fruit and vegetables of 500g/day, a stark contrast to the 6% in RMA. Median dietary fibre intake for the SCD was 26g/d, (IQR 21-33), compared to 16g/d (IQR 12-22) in RMA. Elevated saturated fatty acid (SFA) intake was observed in both groups, with the SCD group also consuming high amounts of red meat. Calcium was the sole nutrient for which the standard diet surpassed the SCD, as 9 out of 10 participants had inadequate intake. While children on the SCD showed a lower likelihood of nutrient inadequacy compared to the general population, inadequate calcium intake and elevated SFA and red meat consumption are concerning given known comorbidities in JIA. These results highlight the importance of disease-specific dietary guidance to ensure optimal support for patients and parents.

## Graphical abstract

**Figure:**
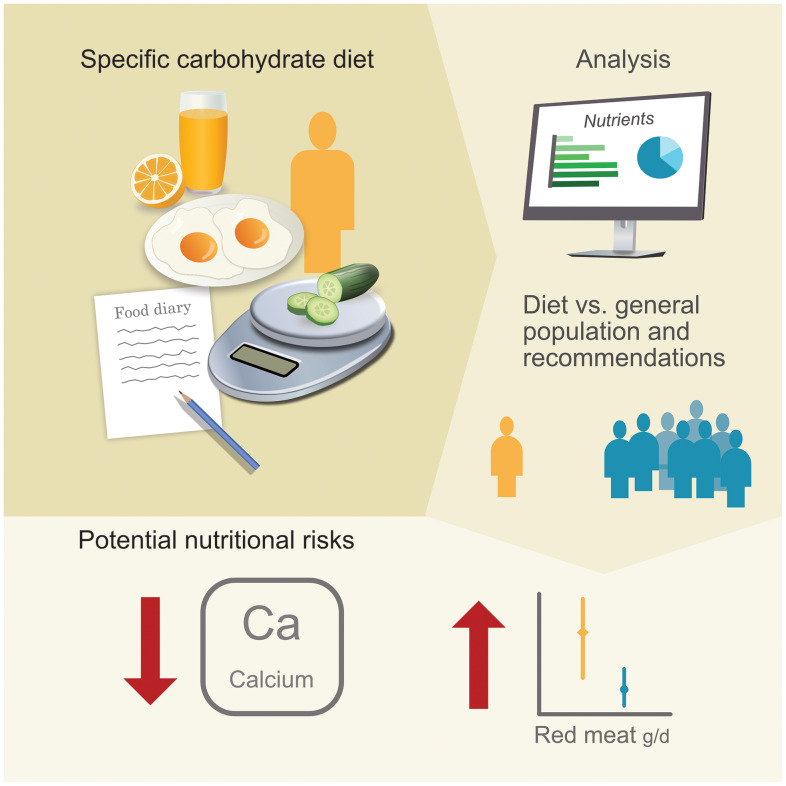

## Introduction

Juvenile idiopathic arthritis (JIA) is an autoimmune disease affecting approximately two million children globally^(1)^. It causes joint inflammation and damage, resulting in pain, morning stiffness, swelling, tenderness, and limited mobility^(2)^. Environmental factors are thought to be implicated in the development of JIA^(3,4)^. Despite advances in immunosuppressive therapies, significant challenges remain, as many patients experience treatment failure and persistent symptoms even with well-managed disease^(5–8)^. This highlights the need for additional interventions in JIA management.

Diet has been identified as a primary research focus for JIA, as indicated by patients, caregivers, and healthcare professionals^(9)^. A recent survey of 261 parents revealed that one in three families had independently experimented with exclusion diets to address their child’s illness^(10)^. Implementing unproven exclusion diets without the involvement of healthcare providers may, however, pose risks to the child, such as weight loss and nutritional deficiencies^(11)^.

Despite a large interest from affected families and clinicians, the number of studies of dietary interventions in JIA is limited and most have focused on specific nutrients or dietary supplements^(12–14)^. Considering the complexity of dietary patterns and foods, where nutrients and bioactive compounds probably interact in ways we do not yet understand, several studies have suggested that research should focus on evaluating the effects of whole diets rather than individual foods or nutrients^(15–17)^.

To our knowledge, only two dietary interventions with whole diets have been investigated for potential anti-inflammatory effects in JIA: exclusive enteral nutrition^(18)^ and the specific carbohydrate diet (SCD)^(19)^. The studies were small, but reported a notable anti-inflammatory impact on active joints, reduced morning stiffness and pain, and a significant decrease in several inflammatory proteins^(18,19)^.

The SCD is an exclusion diet that has been popularised for use in inflammatory bowel disease (IBD) with promising results shown in numerous studies^(20–26)^. It eliminates all grains, added sugar, most processed foods, and dairy products, except for hard cheese and yoghurt. Although the SCD provides a structured framework, it can be challenging to follow^(27)^, which may result in a dietary intake that differs from that intended. However, research on dietary intake while following the SCD is limited, with only one study involving eight children with IBD which reported adequate energy and micronutrient intakes except for vitamin D and calcium^(28)^. Given the limited research on the potential nutritional risks of the SCD across different populations and the increasing interest in dietary interventions in paediatric rheumatology, it is crucial to enhance our understanding of the nutritional implications associated with the SCD in children with JIA.

Therefore, this study aims to describe and evaluate the SCD’s impact on both food and nutrient intakes in children with JIA and to explore how this compares to intakes in the general population and to nutrition recommendations.

## Methods and materials

### Study design

In this study we analysed dietary data collected in a previous intervention study conducted between 2017 and 2020, exploring the anti-inflammatory effects of the SCD in children with JIA^(19)^.

### Participants

In addition to the inclusion and exclusion criteria applied in the original study^(19)^, participants had to have provided a three-day dietary record while strictly following the SCD. Of the 22 children included in the original study, 12 fulfilled the above criteria. Incomplete diet records (e.g. missing portion sizes or amount of food item) or records containing food items not allowed on the SCD led to exclusion. Of the 12 eligible participants, one was excluded due to an incomplete food record and one due to starting oral nutrition supplements.

### Dietary intervention

Before inclusion, the families participated in a teaching session with the study dietitian. Participants were instructed to introduce the change towards the SCD in a stepwise manner over a period of two weeks and then to keep to the SCD strictly for a minimum of four weeks. The families were supplied with a recipe booklet developed specifically for the study, lists of the foods to include/exclude, as well as a list of SCD-compliant pre-packaged food items available in local supermarkets. A dietitian and a physician were available for support throughout the intervention and participants were encouraged to contact them with any questions they had regarding the diet or the study. Towards the end of the four-week intervention, participants kept a 3-day food diary to assess intake and compliance. Participants were instructed to write down everything consumed over two weekdays and one day during the weekend, estimating the amounts by weighing or using common household measurements, such as litres, decilitres, tablespoons, and teaspoons. In addition, they were asked to supply a detailed description of the food items, for example, brand name or percentage of fat in the product consumed. Post analysis, the study dietitian gave each participating family feedback on the child’s food diary. The feedback included subjects such as fat quality, diet variety, and weight loss prevention strategies. A more detailed description of the methods and intervention have been published elsewhere^(19)^.

### Reference data

The latest Nordic Nutrition Recommendations (NNR) from 2023 were used for reference values for foods, macronutrients (the percentage of energy intake (%E)), including fats (total, saturated fatty acids (SFA), monounsaturated fatty acids (MUFA), polyunsaturated fatty acids (PUFA), and omega-3 polyunsaturated fatty acids), carbohydrates, and protein, as well as the average requirement (AR) of selected micronutrients. The micronutrient adequacy was assessed for each child using the AR corresponding to their respective age and gender. This was then presented as the proportion of participants with an intake below AR, which is estimated to cover the nutrient requirement for approximately half of the individuals in a population. An intake below AR signifies a relatively high probability of inadequate intake^(29)^.

The SCD results were put into context using data from the Swedish national dietary survey Riksmaten Adolescents (RMA), which was performed during 2016–2017^(30)^. This data was obtained from the Swedish Food Agency upon request and included a nationally representative sample of 3099 children and young adults between the ages of 10-21 years. Dietary intake data were collected using a validated digital method based on 24h recall called RiksmatenFlexDiet, developed especially for the RMA^(31)^. More detailed descriptions of the methods have been published elsewhere^(30)^. To better match the SCD cohort, we excluded 209 young adults >17 years in our analysis of RMA and used only the registrations deemed valid according to the misreporting analyses performed for the RMA report^(32)^.

### Diet analyses

In this study, we present the short-term food intake from two (RMA) or three days (SCD). To ensure uniform input and the quality of the analysis, the first author and dietitian, NH, controlled each analysed record which had been previously entered by the study dietitian and performed necessary adjustments for uniformity. No dietary supplements were taken by the participants while adhering to the SCD. Food items were sorted into groups, first to match the food groups in RMA, with some later combined to form a final 17 groups (Supplementary Table 1). Intakes of energy (MJ or kJ/day), macronutrients (g/day, %E) and micronutrients (μg/day, mg/day, RE/day) were calculated from food intakes reported in the three-day diet records (SCD) using the nutritional analysis programme DietistNet (Kost och Näringsdata, Bromma, Sweden). Corresponding items from the Swedish Food Agency’s food database were used; however, where food items were missing, data were supplemented using other countries’ or producer’s databases, also found in DietistNet. Since no data on physical activity were collected, each individual’s energy intake was compared to an estimation of their energy expenditure calculated using the equations by Henry^(33)^ multiplied by a physical activity level (PAL) factor corresponding to low physical activity for the respective ages^(34)^. The results were compared to their respective weight change during the four weeks on the diet to assess implausible energy intakes.

### Clinical and laboratory data

Clinical data, including height, weight and blood samples, were collected at inclusion and at four weeks. Disease activity was measured using the Juvenile Arthritis Disease Activity Score (JADAS27), which comprises a joint count (0–27 active joints), patient-reported global assessment of well-being on a visual analogue scale (VAS) (0 – 10 cm) (assessed by a parent if the child is ≤ 9 years old), physician’s global assessment of disease activity on a VAS (0 –10 cm), and normalised erythrocyte sedimentation rate ((ESR in mm/h - 20)/10) on a scale (0–10) with a maximum total score of 57^(35)^. Serum 25-hydroxy Vitamin D was measured in all ten children with JIA using an immunochemistry assay. The following reference values for the assessment of vitamin D status were used: <30nmol/l risk for deficiency, <50nmol/l risk for inadequate status^(29)^.

### Statistical analyses

All analyses were carried out using R Statistical Software version 4.3.2 (R Foundation for Statistical Computing, Vienna, Austria) and figures were produced using the ggplot2 package. Due to the small sample in the SCD group, descriptive statistics and non-parametric tests were used, and the significance level was set at 0.05. Median (Md) and interquartile range (IQR) were calculated for total energy, macronutrients, micronutrients, and for selected food items. Weight change during the SCD intervention was presented as weight change %. BMI z-scores were calculated using WHO’s anthroplus R package^(36)^ For the drop-out analysis, we employed the Independent-Samples Mann-Whitney U test for all variables except gender, for which we used Pearson’s chi-square test. We further calculated the proportion of children from SCD and RMA with an intake below average requirement (AR) levels according to the NNR 2023 for selected micronutrients. Where possible, a statistical method calculating z-score statistics was used to assess the probability of the usual intake while following the diet being adequate or inadequate based on the reported individual intake on the SCD^(37)^.

### Ethical approval and consent to participate

This study was conducted according to the guidelines laid down in the Declaration of Helsinki and all procedures involving human subjects/patients were approved by the regional ethics committee in Uppsala County (Dnr 2016/263) and received additional approval from the Swedish Ethical Review Authority (Dnr 2020-01494). Written informed consent was obtained from all parents and from children aged twelve years or older.

## Results

### Study participants

A total of ten children with JIA submitted complete food records during the SCD intervention. The median age was 11.4 years for SCD and 14.4 years for the RMA cohort. The demographic data of the SCD participants are presented in Table 1.

**Table 1.** Clinical characteristics of ten children with juvenile idiopathic arthritis treated with the specific carbohydrate diet for four weeks

ID	Gender	ILAR category	Age at inclusion, years	Duration of JIA, years	Medical treatment during intervention	BMI at inclusion	BMI-for-age Z-score^a^	Weight change during intervention (%)	Serum-level of vitamin-D at inclusion (nmol/L)	Serum-level of vitamin-D at 4 weeks (nmol/L)
A	M	Oligo pers	9.0	6.1	No	17.9	+1	–2.1	52.1	36.1
B	F	Oligo pers	11.7	8.5	Abatacept + MTX	16.55	–0.56	+0.3	54.9	50.4
C	M	Poly RF-	17.1	3.4	MTX	21.21	+0.01	–1.6	34.4	28.3
D	F	Oligo pers	14.4	3.6	Etanercept + MTX	17.15	–1.15	+1.5	87.4	74.0
E	F	Oligo pers	11.24	7.6	Infliximab + MTX	15.37	–1.07	–0.6	55.5	55.3
F	F	Poly RF+	11.2	2.4	Adalimumab + MTX	20.15	+1.03	–1.2	41.6	58.1
G	M	Poly RF-	12.3	3.3	No	26.26	+2.43	–2.3	44.0	37.2
H	F	Oligo pers	6.4	0.3	MTX	13.65	–1.17	+5.8	missing	71.0
I	M	Oligo pers	12.5	9.3	Adalimumab + MTX	21.01	+1.2	–4.5	69.6	54.4
J	F	Oligo ext	8.0	6.7	Tozilicumab	14.94	–0.44	–4.5	67.7	37.5

ILAR, International League of Associations for Rheumatology; JIA, juvenile idiopathic arthritis; oligo pers, oligoarticular persistent; MTX, methotrexate; poly RF-, polyarticular rheumatoid factor negative; polyarticular rheumatoid factor positive; oligo ext, oligoarticular extended.

a WHO, World Health Organization BMI-for-age Z-score cut-off points: < − 2.0 = thinness, > + 1.0 = overweight, > + 2.0 = obesity.

To assess potential selection bias, we compared the characteristics of included participants (n = 10) with those who did not submit complete food records (n = 12). No statistically significant differences were found between the groups for the following variables. The median age at inclusion for participants was 11.4 years (IQR: 8.7–13.0) compared to 14.0 years (IQR: 10.5-16.6) for drop-outs (p = 0.38). Of the participants, 60% were girls, while 83% of the drop-outs were girls (p = 0.22). The BMI z-score was also similar, with a median of −0.22 (IQR: −1.1 to 1.1) for participants and −0.11 (IQR: -0.5 to 1.3) for drop-outs (p = 0.58). Additionally, the change in disease activity (JADAS27) after one month of SCD showed a median of -2.3 (IQR: −5.2 to −1.2) for participants compared to −2.1 (IQR: −3.9 to 0.1) for drop-outs (p = 0.54).

### Food intake

**Figure 1.**
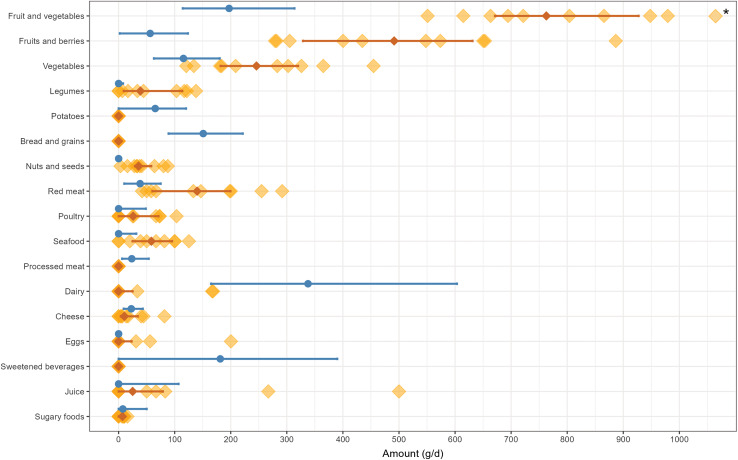
Food intakes of ten children and adolescents with juvenile idiopathic arthritis adhering to the specific carbohydrate diet (SCD) and a general population sample (*n* = 1282) from ‘Riksmaten Adolescents 2016-17’ (blue circles and lines). The intakes are presented individually for the children on the SCD (orange squares and lines) and with median and IQR for both groups. * = fruit and vegetable intake of 1300 g.

The food intakes of the children on the SCD and RMA differed (Fig. 1). Overall, the SCD group had a high consumption of fruit, vegetables, nuts, seeds, legumes, eggs, juice, seafood, red meat, and poultry. Fruit and vegetables constituted a substantial portion of the intake for the SCD participants, ranging from 550 to over 1300 g/d (Md 763 g/d, IQR 671–927); all reached the minimum recommended intake of 500 g/d. However, in RMA the median intake was approximately 200 g/d (IQR 114–314), with only 6% reaching the minimum recommendations. The children on the SCD also consumed more fruit than vegetables, while the opposite was observed in RMA. Moreover, while adhering to the SCD, the children had no intake of processed meat, bread, grains, potatoes, or sweetened beverages and a lower consumption of sugary foods, which primarily consisted of honey. Milk and yoghurt were only consumed by 3/10 individuals while 8/10 ate cheese. Two of the children did not consume any dairy products. In contrast, RMA participants consumed substantial amounts of dairy products (IQR 165–603 ml/d) and sweetened beverages, while the median intake was close to zero for eggs, legumes, nuts, and seeds.

### Energy intake and macronutrient distribution

**Figure 2.**
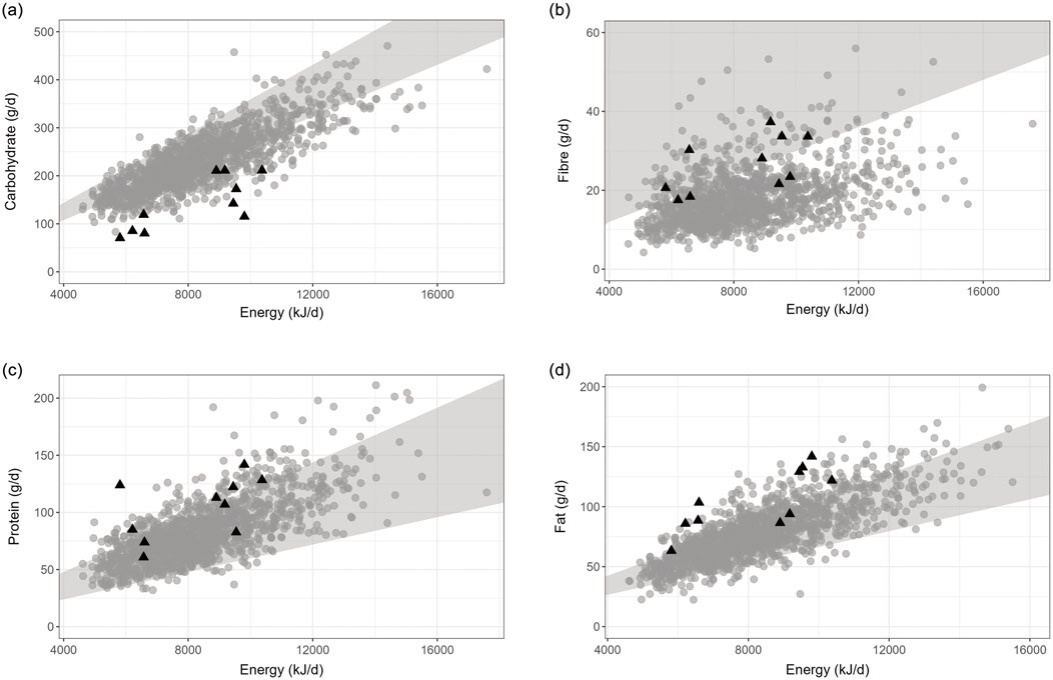
Levels of macronutrient intake in relation to energy in kilojoules (kJ) in ten children with juvenile idiopathic arthritis on the specific carbohydrate diet (black triangles). Reference data from 1282 children from the general population, Riksmaten Adolescents, are presented in grey circles and reference intervals from the Nordic Nutrition Recommendations (NNR 2023) in the grey area. (a) Intake of carbohydrate, recommended intake 45–60 %E (b) Intake of fibre, recommendation calculated based on 3 grams/MJ (c) Intake of protein, recommended intake 10–20 %E (d) Intake of fat, recommended intake 25–40 %E.

Participants following the SCD exhibited an average daily energy intake ranging from 5.8-10.4 MJ, comparable to both the general population (4.6–17.5 MJ) and the NNR reference interval (5.9–12.7 MJ) for the ages concerned. However, several children had an energy intake slightly below their individual estimated energy requirement which also coincided with some weight loss (Table 1). Specifically, children with a higher initial weight at inclusion, including those with overweight and obesity, lost weight during the intervention, while others with a lower body weight at inclusion tended to gain weight. In terms of macronutrient distribution, the median energy percentages deviated from the reference values for SCD participants. As illustrated in Fig. 2(a), children in the general population (RMA) and those on the SCD had a carbohydrate intake at the lower end or below the recommended range of 45–60 %E. Notably, children on the SCD had low carbohydrate intakes, with energy from carbohydrates ranging from 20–40 %. Furthermore, six out of ten children met the minimum recommended fibre intake of 3 g/MJ, with an intake ranging between 18–37 g/d (Md 26 %E, IQR 21–33) as depicted in Fig. 2b. Two of the four individuals with a recorded fibre intake below the recommendation were close to meeting it. Conversely, the majority of children from the RMA group had a documented fibre intake below 20 g, irrespective of their energy intake, with a median intake of 17g (IQR 13–22). Furthermore, as illustrated in Fig. 2(c), six of the ten children on the SCD had a higher protein intake than recommended, (Md 21 %E, IQR 19–23). In RMA, very few had an intake below the recommendation 10–20 %E, and most had an intake within the recommended interval (Md 16 %E, IQR 14–19). Only a few individuals from the RMA group had a fat intake below the recommended range of 25–40 %E, with the majority consuming fat at the upper end of the range or exceeding it. The median fat intake was 35 %E (IQR 31–40). This trend was similarly observed in the recorded fat intake on the SCD, where eight out of ten individuals consumed fat above the recommended levels or at the upper limit of the recommended range (Md 51 %E, IQR 42–52).

### Fat intake

**Figure 3.**
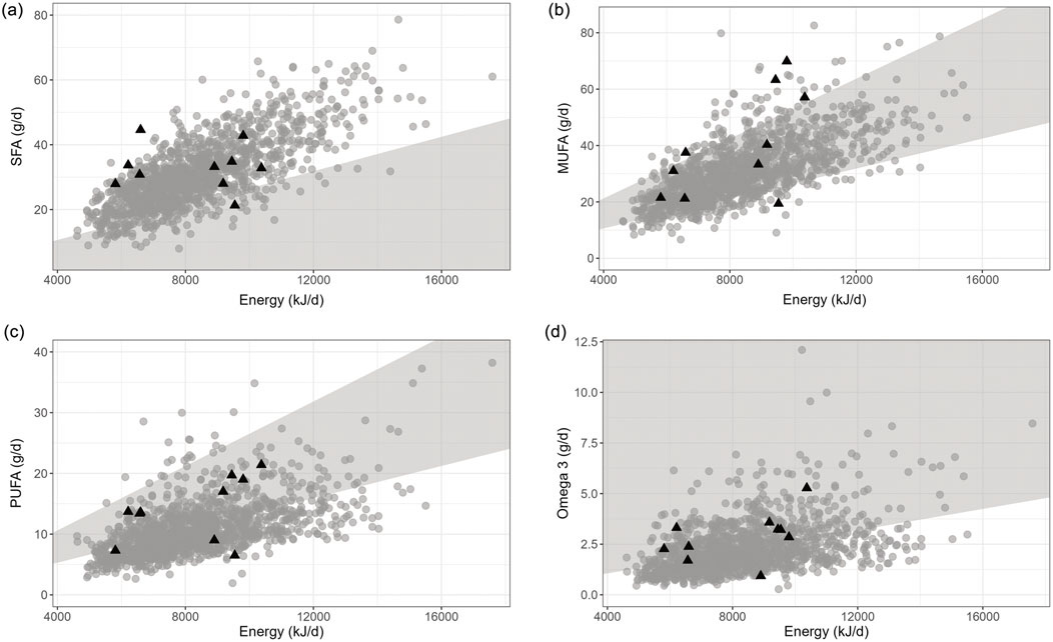
Intake of fatty acids in relation to energy in kilojoules in ten children with juvenile idiopathic arthritis on the specific carbohydrate diet (black triangles). Reference data from 1282 children from the general population, Riksmaten Adolescents, are presented in grey circles and from the Nordic Nutrition Recommendations (NNR 2023) in the grey area. (a) Intake of saturated fatty acids, recommendations <10 %E (b) Intake of monounsaturated fatty acids, recommended intake 10–20 %E (c) Intake of polyunsaturated fatty acids, recommended intake 5–10 %E (d) Intake of omega 3 fatty acids >1 %E.

MUFA intake was consistent for both the RMA and the SCD, with the majority of children adhering to the recommended range of 10–20 %E (Fig. 3(a)); the SCD had an intake of Md 18 %E (IQR 14–21), the RMA Md 13 %E (IQR 11-16). Regarding PUFA intake, illustrated in Fig. 3(b), seven out of ten children on the SCD reported levels within the recommended range of 5–10 %E (Md 8 %E, IQR 5–8). Conversely, most children in the RMA group had a lower intake of PUFAs (Md 5 %E, IQR 4–6) compared to recommendations. Furthermore, the proportion of energy derived from SFAs exceeded recommendations in both groups (Fig. 3(c)), with an IQR of 12–18 %E for the SCD and 12–16 %E for the RMA, which should ideally be maintained below 10 %E. Although the SFA intake was similar between the SCD and RMA, it was proportionally greater in the RMA due to a lower amount of energy coming from fat (31–40 %E) compared to the SCD (42–53 %E). Regarding omega-3 intake, children on the SCD had a median intake of 1.32 %E (IQR 1.14–1.47), most aligning with the recommended level of 1 %E, while those in the RMA group had a median intake of 0.89 %E (IQR 0.69–1.2), with many falling below the recommended threshold.

### Micronutrient intake

**Figure 4.**
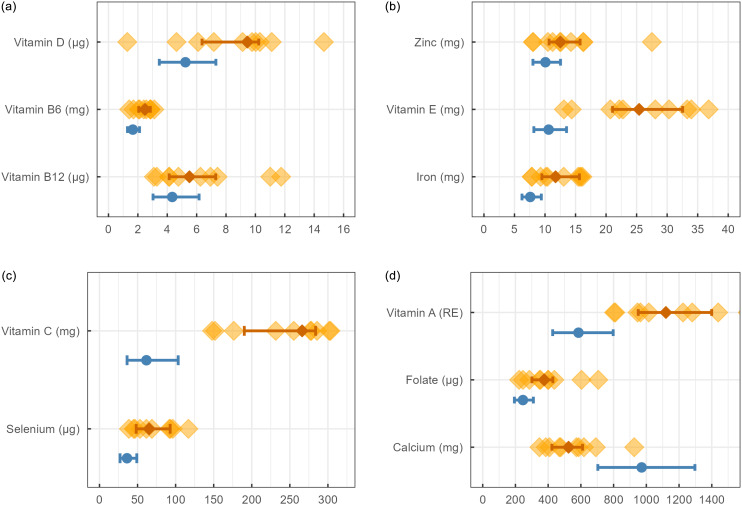
Micronutrient intakes in ten children with juvenile idiopathic arthritis (JIA) on the specific carbohydrate diet (SCD) and a general population sample (n = 1282) from ‘Riksmaten Adolescents 2016-17’ (in blue, median=circles and IQR=lines). The intakes are presented individually for the children on the SCD (orange diamonds and lines) and with median and IQR for both groups. (a) Intakes of vitamin D, B6 and B12, (b) Intakes of zinc, vitamin E and iron, (c) Intakes of vitamin C and selenium, (d) Intakes of vitamin A, folate and calcium.

The micronutrient intakes of children on the SCD and the group from RMA are illustrated in Fig. 4., and the proportion of individuals with an intake below AR and thus an increased risk of inadequate intake, is presented in Table 2. For five of the micronutrients (vitamin A, B6, B12, C, E, and folate), none of the children on the SCD had an intake below AR, compared to approximately 20–80% in the general population. Calcium was the only nutrient for which a standard diet provided a higher intake than the SCD since 90% of participants were below the AR (Table 2). Individual z-score calculations revealed a probability of the usual calcium intake while adhering to the SCD being inadequate, ranging from 50-98% (Supplementary Table 2). Furthermore, 40% of those on the SCD and 77% of the RMA group had an intake of vitamin D lower than the AR. Serum samples for the SCD group showed, however, that only one child had a confirmed inadequate Vitamin D status at the four-week follow-up (S-25(OH)D = 28.5 nmol/L) (Table 1).

**Table 2.** Proportion of children with micronutrient intakes below reference values: comparison between patients with juvenile idiopathic arthritis (JIA) on specific carbohydrate diet (SCD) and general population

Nutrient	SCD (*n* = 10) < AR^a^ (%)	RMA (*n* = 1282) < AR^a^ (%)
Vitamin A (RE)	0	44
Vitamin D (µg)	40	77
Vitamin E (mg)	0^b^	73^b^
Vitamin B_6_ (mg)	0	19
Folate (µg)	0	32
Vitamin B_12_ (µg)	0^b^	25^b^
Vitamin C (mg)	0	80
Calcium (mg)	90	51
Iron (mg)	10	52
Zinc (mg)	10	50
Selenium (µg)	10^b^	78^b^

AR, average requirements; RMA, Riksmaten Adolescents (2016-17); RE, retinol equivalents.

a Proportion of children below average requirement (AR) according to Nordic Nutrition Recommendations (NNR 2023).

b Based on provisional AR = adequate intake x 0.8, assuming CV is 12.5%. The true AR is probably lower.

## Discussion

This study presents insights into the impact of food choices within the framework of the SCD in 10 children with JIA. It also sheds light on the distribution of macronutrients, the risk for inadequate micronutrient intake, and how intakes relate to those of the general population.

As expected, the distribution of macronutrients among the participants deviated from general recommendations due to the exclusion of grains in the SCD, which led to their substitution through increased intakes of vegetables, legumes, nuts, and seeds, known for their nutrient density, fibre content, and anti-inflammatory phytonutrients, such as polyphenols^(38)^. While high in nutrients, fruit and vegetables are generally low in energy, which together with the exclusion of sweetened beverages, sweets and fast foods may explain the weight loss seen in some participants. Even though this occurred mostly in children with a higher initial weight, it should be monitored by a dietitian, which was the case in this study. Interestingly, these results differ from those of children with IBD where most gained weight while on the SCD^(28)^. One plausible explanation for this difference could be an enhanced absorption of nutrients linked to reduced intestinal inflammation in children with IBD after adopting the SCD.

In addition, we saw a high intake of unprocessed animal-based protein, from sources such as eggs, seafood, meat, and poultry, and consequently an elevated intake of SFAs and red meat compared to recommendations. Despite an elevated intake of SFAs in both groups, the distribution reported in this study suggests intakes with a slightly better fat quality in the SCD group compared to the RMA, which exhibited a proportionally higher intake of SFAs. Nevertheless, a reduction in SFAs may be particularly important for children with JIA who face an increased risk of metabolic syndrome and cardiovascular disease in adulthood^(29,39–46)^.

Most children following the SCD showed a micronutrient intake well above the AR, suggesting a low probability of inadequate intake. However, low calcium intake, probably due to limited consumption of dairy or fortified alternatives, is concerning given that JIA can affect bone health, with reduced bone mineral density and increased fragility fracture risk^(47)^. This underscores the importance of optimising calcium and vitamin D intake for these children. Despite the low dairy intake, only 40% of the children following the SCD had a risk of inadequate vitamin D intake, compared to 77% of children in the RMA. This disparity may be attributed to the relatively high intake of seafood among SCD followers, which serves as a source of vitamin D^(48)^. In the study by Braly et al., all children adhering to the SCD had an inadequate vitamin D intake, but their reference value is set at 15 μg compared to 10 μg in the Nordic countries^(28,29)^. It is important to note that exposure to sunlight contributes a significant amount of vitamin D. Although 4/10 following the SCD were found to be at risk of inadequate intake, only one participant had a low status confirmed. Similarly, of 1100 tested in the RMA cohort, only 10% had a low status of vitamin D^(32)^.

The primary limitation of this study is its small sample size, which impacts the range of analyses that could be conducted. However, the small sample size also allowed for high quality, uniformity, and control during the dietary data entry and analysis process. Due to variations in intake (day-to-day and seasonal), this result may not reflect the children’s usual year-round intake^(49,50)^. To strengthen the results, we calculated the probability of their individual usual intake being adequate, which confirmed for example the inadequate calcium intake. Another significant limitation was the high exclusion rate. Although the drop-out analysis did not reveal any differences between participants who submitted diet records and those who did not, selection bias cannot be ruled out as the sample size may have been insufficient to detect existing differences between these groups. Furthermore, due to the lack of specific nutrient recommendations for children with JIA, we used values for healthy children, even though the participants had a chronic inflammatory disease which could affect their nutrient requirements, especially during flares with increased inflammation^(51)^. In this study, the impact of this is thought to be minimal, since a majority of the children with JIA had low disease activity. However, further work is needed to establish specific nutrient recommendations for children with chronic inflammatory diseases, which could be a step towards better disease management. Despite promising results from the SCD^(19)^, future studies should investigate if a reduced intake of SFAs and red meat could potentially yield even better anti-inflammatory results for children with JIA, as well as lower their risk of future comorbidities.

## Conclusion

The dietary assessment of 10 children following the SCD revealed that the diet offered a significant intake of fruit, vegetables and dietary fibre. Despite the exclusion of specific food groups, the likelihood of inadequate intake for most nutrients seems to be lower than in the general population, except for calcium. Other nutritional risks identified while adhering to the SCD include elevated intakes of SFAs and red meat, both of which are concerns particularly relevant for children with JIA. These results highlight the importance of monitoring and tailored dietary guidance, specific to the disease for optimal patient and parent support.

## Supporting information

Hagström et al. supplementary material 1 Hagström et al. supplementary material

Hagström et al. supplementary material 2 Hagström et al. supplementary material

## Data Availability

The data that support the findings of this study are available from the corresponding author, upon reasonable request.

## Supplementary materials

The supplementary material for this article can be found at https://doi.org/10.1017/jns.2024.92
