# Supplementary material for: Exploring nutritional risks of the specific carbohydrate diet: food and nutrient intake in children with juvenile idiopathic arthritis
Source: J Nutr Sci. 2025 Jan 23;14:e9. doi: 10.1017/jns.2024.92 (PMC11811854; doi:10.1017/jns.2024.92)
Supplement: Hagström et al. supplementary material 1 — Hagström et al. supplementary material [file S2048679024000922sup001.docx]

| Supplementary table 1. Content and categorization of food groups. | | |
| --- | --- | --- |
| Food groups | Included foods | Excluded foods |
| Fruits and vegetables | Fruits, berries, legumes and vegetables | Potatoes and mushrooms |
| Vegetables | Leafy greens and root vegetables | Potatoes, legumes, mushrooms |
| Fruits and Berries | All fruits and berries, including frozen, dried, canned and fresh |  |
| Legumes | Beans, lentils etc. |  |
| Potatoes | Potatoes |  |
| Red meat | Beef, lamb, pork etc. | Processed meat |
| Processed meat | Meat or poultry products containing nitrate or preservatives, for example, sausage and ham |  |
| Poultry | Poultry | Processed poultry |
| Seafood | Fish and seafood, such as shrimp, crab, crayfish etc. | Caviar, fish roe |
| Eggs | Including eggs from composite dishes where eggs are the main ingredient, such as omelettes |  |
| Nuts and seeds | All nuts and seeds, including coconut |  |
| Dairy | Milk, fermented milk and yoghurt | Cheese, cream, crème fraiche, butter |
| Cheese |  |  |
| Breads and grains | Bread, crispbreads, crackers, bulgur, breakfast cereals, flour, pasta and rice |  |
| Sugary foods | Sweets, chocolate, honey, syrup and sugar |  |
| Sweetened beverages | Soft drinks, chocolate drinks, sports drinks, squashes and cordials |  |
| Juice | Unsweetened fruit juice |  |
