# Supplementary material for: Exploring nutritional risks of the specific carbohydrate diet: food and nutrient intake in children with juvenile idiopathic arthritis
Source: J Nutr Sci. 2025 Jan 23;14:e9. doi: 10.1017/jns.2024.92 (PMC11811854; doi:10.1017/jns.2024.92)
Supplement: Hagström et al. supplementary material 2 — Hagström et al. supplementary material [file S2048679024000922sup002.pdf]

Supplementary table 2. Micronutrient intake from diet alone in children with juvenile idiopathic arthritis following the specific carbohydrate diet (SCD) and in the general population (RMA).

Nutrient	ID	Observed intake	Observed intake < AR[a]	Z-score	Z-score conclusion	Probability of correct conclusion	SCD Median intake (IQR)	RMA Median intake (IQR)	Proportion of RMA <AR[a]
Vitamin A (RE)[b]	A	1280.65	no	NA[c]	NA	NA	1119.6 (950.7-1399.4)	585.3 (427.7 - 797.5)	34%
	B	1224.1	no						
	C	1619.87	no						
	D	946.22	no						
	E	1711.11	no						
	F	963.94	no						
	G	1439.03	no						
	H	1015.15	no						
	I	803.08	no						
	J	810.44	no						
Vitamin D (µg)	A	10.02	no	NA	NA	NA	9.5 (6.4-10.2)	5.2 (3.5 - 7.3)	77%
	B	11.11	no						
	C	14.66	no						
	D	9.77	no						
	E	10.3	no						
	F	7.17	yes						
	G	1.28	yes						
	H	6.1	yes						
	I	9.13	no						
	J	4.63	yes						
Vitamin E (mg)[d]	A	13.09	no	2.44	Adequate usual intake	0.98	25.4 (21-32.5)	10.6 (8.2 - 13.5)	27%
	B	36.8	no	20.1		0.98			
	C	33.28	no	12.17		0.98			
	D	33.96	no	17.9		0.98			
	E	22.77	no	9.58		0.98			
	F	20.7	no	8.03		0.98			
	G	22.16	no	7.29		0.98			
	H	28.06	no	20.06		0.98			
	I	30.3	no	12.2		0.98			
	J	14.37	no	5.37		0.98			
Vitamin B_6 (mg)	A	2.4	no	2.59	Adequate usual intake	0.98	2.5 (2-2.9)	1.7 (1.3 - 2.1)	19%
	B	3.1	no	4.83		0.98			
	C	2.84	no	2.44		0.98			
	D	2.85	no	8.6		0.98			
	E	2.12	no	2.44		0.98			
	F	1.7	no	1.44		0.98			
	G	2.57	no	2.66		0.98			
	H	2.04	no	4.1		0.98			
	I	2.88	no	3.19		0.98			
	J	1.41	no	1.72		0.98			
Folate (µg)	A	285.19	no	1.3	Adequate usual intake	0.85	375.9 (300.8-428.7)	245.9 (193.7 - 309.8	33%
	B	603.9	no	5.2		0.98			
	C	399.06	no	1.38		0.85			
	D	706.31	no	5.84		0.98			
	E	246.28	no	0.71		0.7			
	F	352.75	no	2.05		0.98			
	G	347.67	no	1.59		0.93			
	H	401.17	no	4.69		0.98			
	I	437.88	no	2.45		0.98			
	J	222.62	no	1.36		0.85			
Vitamin B_{12} (µg)[d]	A	6.26	no	NA	NA	NA	5.5 (4.1-7.3)	4.3 (3 - 6.2)	20%
	B	6.94	no						
	C	7.42	no						
	D	11.01	no						
	E	4.76	no						
	F	4.13	no						
	G	3.29	no						
	H	3.09	no						
	I	11.75	no						
	J	4.13	no						
Vitamin C (mg)	A	301.6	no	NA	NA	NA	266.1 (190.2-283.9)	61.7 (36.2 - 103.4)	54%
	B	278.21	no						
	C	285.79	no						
	D	303.33	no						
	E	147.84	no						
	F	231.49	no						
	G	255.19	no						
	H	277.08	no						
	I	176.41	no						
	J	151.72	no						
Calcium (mg)	A	618.37	yes	-0.19	Inadequate usual intake	0.5	524.7 (423-610.4)	971.4 (703.6 - 1297)	51%
	B	572.5	yes	-1.72		0.85			
	C	468.36	yes	-1.66		0.95			
	D	476.83	yes	-2.12		0.98			
	E	346.68	yes	-2.67		0.98			
	F	586.47	yes	-1.66		0.85			
	G	407.85	yes	-1.86		0.85			
	H	385.49	yes	-1.7		0.85			
	I	925.94	yes	-0.18		0.5			
	J	691.04	no	0.08		0.5			
	A	7.84	no						
	B	15.7	no						
	C	13.06	no						

Iron (mg)	D	15.44	no	NA	NA	NA	11.7 (9.5-15.6)	7.6 (6.2 - 9.4)	68%
	E	10.13	no						
	F	7.83	yes						
	G	16.13	no						
	H	9.27	no						
	I	15.88	no						
	J	10.39	no						
Zinc (mg)	A	10.47	no	0.87	Adequate usual intake	0.7	12.5 (10.7-15.8)	10.1 (8 - 12.5)	39%
	B	16.3	no	2.8		0.98			
	C	11.25	yes	-0.07		0.5			
	D	12.45	no	0.94		0.85			
	E	8.04	no	0.04		0.5			
	F	12.58	no	1.56		0.93			
	G	16.25	no	1.69		0.95			
	H	8.04	no	1.68		0.95			
	I	27.5	no	4.09		0.98			
	J	14.24	no	4.9		0.98			
Selenium (µg)	A	61.55	no	NA	NA	NA	65.2 (48.3-92.8)	36.2 (27 - 49)	75%
	B	91.8	no						
	C	93.18	no						
	D	116.78	no						
	E	68.83	no						
	F	53.18	no						
	G	38.62	yes						
	H	46.61	no						
	I	96.28	no						
	J	44.83	no						

[a] Children with intake below average requirement (AR) according to Nordic Nutrition Recommendations (NNR 2023)

[b] RE: retinol equivalents

[c] NA = not applicable due to not fullfilling the requirements of the equation.

[d] Based on provisional AR = adequate intake x 0.8, assuming CV is 12.5%. True AR is likely lower. Z-score calculated using AI = adequate intake.
